# Kilometric sea level changes during the Messinian salinity crisis caused by river erosion and climate

**DOI:** 10.1126/sciadv.ads9752

**Published:** 2025-07-11

**Authors:** Daniel García-Castellanos, Hanneke Heida, Dan V. Palcu, Francesca Bulian, Francisco Sierro

**Affiliations:** ^1^ Consejo Superior de Investigaciones Científicas (CSIC), Geociencias Barcelona (Geo3BCN-CSIC), Barcelona, Spain.; ^2^ GeoEcoMar, National Institute of Marine Geology and Geo-ecology, Bucharest, Romania.; ^3^ Palaeomagnetic Laboratory Fort Hoofddijk, Utrecht University, Utrecht, Netherlands.; ^4^ University of Groningen, Groningen, Netherlands.; ^5^ Universidad de Salamanca, Salamanca, Spain.

## Abstract

The Messinian salinity crisis (MSC) was a short period of isolation of the Mediterranean Sea that caused the precipitation of a million cubic kilometers of salt. The puzzling sedimentary record that formed after this deposition yields conflicting values of the extent of desiccation. Estimations range from a full exposure of most of the Mediterranean seafloor based on shallow fossil fauna found in the abyss to a nearly full Mediterranean scenario as suggested by similar, fresher-water deposits ubiquitous along the coastline: the so-called Lago-Mare formation. Using a landscape evolution model of the drawdown stage constrained with paleoclimate and sediment budgets, we show that the propagation of an erosional wave into the surrounding continents added a gradual sea level rise superimposed on the climatic oscillations of the Mediterranean. This retrogressive river incision along the spillways of the Paratethys and the Pannonian basins also explains the Mediterranean transition to fresher-water conditions during the last stage of the MSC.

## Teaser

Upon desiccation, the Mediterranean level rose because of the erosional capture of surrounding lacustrine basins.

## INTRODUCTION

By the Late Miocene, the Mediterranean Sea had become enclosed between the African and Eurasian tectonic plates, as all but one of the seaways connecting it to the ocean had closed. The severe tectonic constriction of that remaining seaway, combined with the region’s dry climate, caused salinity to rise (*1*, *2*), disrupting the marine ecosystems well before the onset of salt precipitation (*3*, *4*). The Messinian salinity crisis (MSC) proper started with the onset of massive, widespread gypsum precipitation (*5*) [MSC stage 1, 5.97 to 5.60 Myr ago (million years ago)]. The ongoing tectonic uplift of the seaway eventually blocked the outflow of the deep, high-salinity Mediterranean waters while still allowing the inflow from the ocean, resulting in massive halite precipitation (*6*). Further tectonic uplift lastly blocked this inflow (*1*, *6*), fully landlocking the Mediterranean and setting the stage for a kilometric sea level drawdown by evaporation during stage 2 (5.60 to 5.55 Myr ago). During this stage, about 15% of Earth’s land surface (including the Mediterranean and Paratethyan catchments) became endorheic and hydrologically disconnected from the ocean, and ~5% of the salt dissolved in the oceans was trapped as salt rock in the Mediterranean seafloor.

What happened subsequently is less clear. The Mediterranean became a great system of lakes, the so-called “Lago Mare” (stage 3, 5.55 to 5.33 Myr ago) characterized by the cyclic precipitation of evaporites and deposition of marls (*5*, *7*). While the evaporites are barren of fossil fauna, the marls, especially those deposited toward the end of this stage [sometimes separated as substage 3.2 starting at 5.42 Myr ago (*8*)], are rich in micro- and macrofossils characteristic of brackish-water environments and of Paratethyan origin. The causes for this cyclical transition toward an increasingly less saline and more Paratethys-influenced Lago-Mare (*8*, *9*) remain poorly understood, given the absence of a corresponding coeval climate change (*5*, *10*). Whatever happened during stage 3, it led to a scenario compatible with the abrupt refill with Atlantic waters that formed the present Strait of Gibraltar restored the normal open marine conditions during the Zanclean flood (*11*–*13*) (5.333 Myr ago).

Before and during the deposition of the Lago-Mare deposits (stage 3), the source region for its fauna (the Paratethys) underwent a series of water level fluctuations. After a sea level rise around 6.1 Myr ago (*14*), marked by the invasion of Mediterranean taxa that suggests a double-flow connection between both realms, the Paratethys experienced a series of base-level drops coeval with the MSC (*14*–*16*). The one dated around ~5.4 Myr ago is observed throughout Paratethys (*17*) and led in some cases to hiatuses of around 100 m lasting until the earliest Pliocene (*14*, *16*, *17*), with evidence suggesting a level stagnation toward the end of stage 3 (*18*).

Widespread geophysical and field evidence for erosion of the continental margins around the Mediterranean Sea and for shallow-water Lago-Mare deposits found in boreholes as deep as 2600 m (*19*–*21*) show that sometime during stage 3, the Mediterranean level was at a level of −1.2 km in the western basin (*22*) and perhaps deeper than −2 km in the eastern basin. However, the ubiquity of similar Lago-Mare deposits along the Mediterranean coast (*23*), at elevations close to present sea level in places where tectonic motions are presumed minor, intriguingly points to a concurrent high sea level during the same period. Near Málaga (Alborán Sea), for example, the Lago-Mare facies is found up to 100 m above sea level, and deposition paleodepth is estimated at 50 to 150 m (*24*). In the Mallorca Basin, shallow-water Lago-Mare found up to 70 m above present sea level in areas where tectonic vertical motions should be minor also suggests deposition in a nearly filled Mediterranean with brackish waters populated by Paratethyan ostracods (*22*, *24*, *25*). In addition to this contrast, numerous efforts to constrain the magnitude of the deeper water level markers by isostatically restoring their original depth (*22*, *26*–*29*) have failed to narrow down the drawdown estimations, suggesting that those markers may have actually formed at highly variable levels during MSC stages 2 and 3 (*27*). A recent reanalysis of micropaleontology, sedimentology, and ^87^Sr/^86^Sr ratios at Heraclea Minoa (Sicily) suggests large-amplitude sea level oscillations caused by orbital climatic cyclicity (*30*) during the MSC, although their amplitude seems insufficient to reconcile the shallow-water brackish fauna (*21*) found at very deep drillings within the Deep Sea Drilling Project and Ocean Drilling Program (*19*) with the similarly shallow deposits ubiquitous on the higher margins of the Mediterranean (*31*–*34*). Thus, the problem persists as to how to make a nearly desiccated Mediterranean Sea compatible with a nearly full one during the last stage of the MSC and with the level drop implied by the final flooding.

Because lake outlet incision is a key driver of drainage reorganizations (*35*, *36*), here, we hypothesize that the propagation of an erosional wave into the regions surrounding the desiccated Mediterranean led to a progressive refilling of the basin during stage 3. Erosion along the outlets of the Paratethyan lakes may have caused their drawdown, enhancing the delivery of fresh water to the Mediterranean and explaining the whole range its sea level estimates. To test this hypothesis, we develop a source-to-sink landscape evolution model (LEM) constrained by existing water and sedimentary budgets during the MSC and a paleogeographic reconstruction of the Miocene Mediterranean and Paratethyan region.

## RESULTS

### Model setup

To model topographic and drainage evolution, we make use of TISC (Tectonics, Isostasy, Surface proceses, and Climate) (*37*), an open-source LEM that calculates the steady-flow hydrological balance and erosion rates on a fixed, rectangular finite difference grid. The model adopts a stream power law for fluvial incision and explicitly accounts for the area attained by endorheic lakes formed in local topographic minima to evaporate the water discharge they collect. TISC computes water discharge at each cell in cubic meter per second, disregarding transitory effects such as floods or seasonality. The water sources (runoff from precipitation and from prescribed inputs at the Nile, Volga, and Chad rivers; see Table 1) are instantly balanced by water sinks (lake evaporation and river outputs through the model boundaries). For example, under constant climate conditions (i.e., constant precipitation *P* and evaporation *E*), the total lake area would also remain constant. In such a scenario, if a lake shrinks because of erosion along its outlet, then other lakes must expand further downstream.

**Table 1.** Parameter list of all model runs shown. M1 to M5 are model setups modified from the reference run M0.

Parameter		Reference model M0	Other model setups
Initial time (full isolation, main drawdown)		−5.550 Myr (insolation low)	
Gibraltar opening time		−5.338 Myr (insolation low)	
Final run time		−5.320 Myr	
Temporal resolution: *dt*		0.001 Myr	
Spatial resolution: *dx*, *dy*		10 km	
Evaporation rate at lakes *E*		1.2 m year^−1^ (*66*)	
Transport capacity *K*_t_		1000 kg m^−3^	
Runoff (precipitation) *P*		*P*(*x*, *y*, *z*) = (*P*_0_ + *P_z_z*) (1 – *x* / *P_x_*) (1 – *y* / *P_y_*) × insolation65N / insolation_average	
Runoff at sea level *P_0_*		200 mm year^−1^	M5: 180 mm year^−1^
			Sensitivity test Fig. 5: 0 to 300 mm year^−1^
Runoff dependence with elevation *P_z_*		200 mm year^−1^ km^−1^	M5: 180 mm year^−1^
			Sensitivity test Fig. 5: 0 to 300 mm year^−1^ km^−1^
Runoff dependence with latitude/longitude *P_x_*, *P_y_*		−10,000, 1200 km	
Precession period *P*_per_		(*41*) (fixed to 21.7 kyr for Fig. 5)	
Precession amplitude *P*_amp_		(*41*) (fixed to 0.12 for Fig. 5)	M2: 0
Additional river inputs			
Volga		10,000 m^3^ year^−1^	
Nile		12,799 m^3^ year^−1^	
Chad		1000 m^3^ year^−1^	
Lithospheric elastic thickness *T*_e_		20 km	M4: 10 km
Erodibility bedrock *k*_b_		3.6 × 10^−7^ m year^−1^ Pa^−1.5^	M1: 2.9 × 10^−7^ m year^−1^ Pa^−1.5^
Erodibility sediment		3.6 × 10^−6^ m year^−1^ Pa^−1.5^	M1: 2.9 × 10^−6^ m year^−1^ Pa^−1.5^
			M3: 0 m year^−1^ Pa^−1.5^
			Sensitivity test Fig. 3: 0 to 2 × 10^−5^ m year^−1^ Pa^−1.5^
Density	Fresh water	1000 kg m^−3^	
	Marine water	1015 kg m^−3^	
	Sediment	2000 kg m^−3^	
	Bedrock/crust	2700 kg m^−3^	
	Asthenosphere	3200 kg m^−3^	

The calculated flow of sediment is also conservative, both at the cell scale and at the scale of the entire model domain. The total bedrock erosion matches the increase in sediment volume plus the sediment output through the boundaries at each time step. The rate of erosion/sedimentation is calculated as a power function of water discharge, river channel slope, and erodibility (*37*). The isostatic lithospheric vertical motions caused by changes in the load of water and by erosion/sedimentation are calculated using a thin-plate elastic flexural calculator embedded in TISC. The sensitivity to the elastic thickness is shown through model M4 in fig. S1. The model is described in more detail in Materials and Methods.

**Fig. 1.**
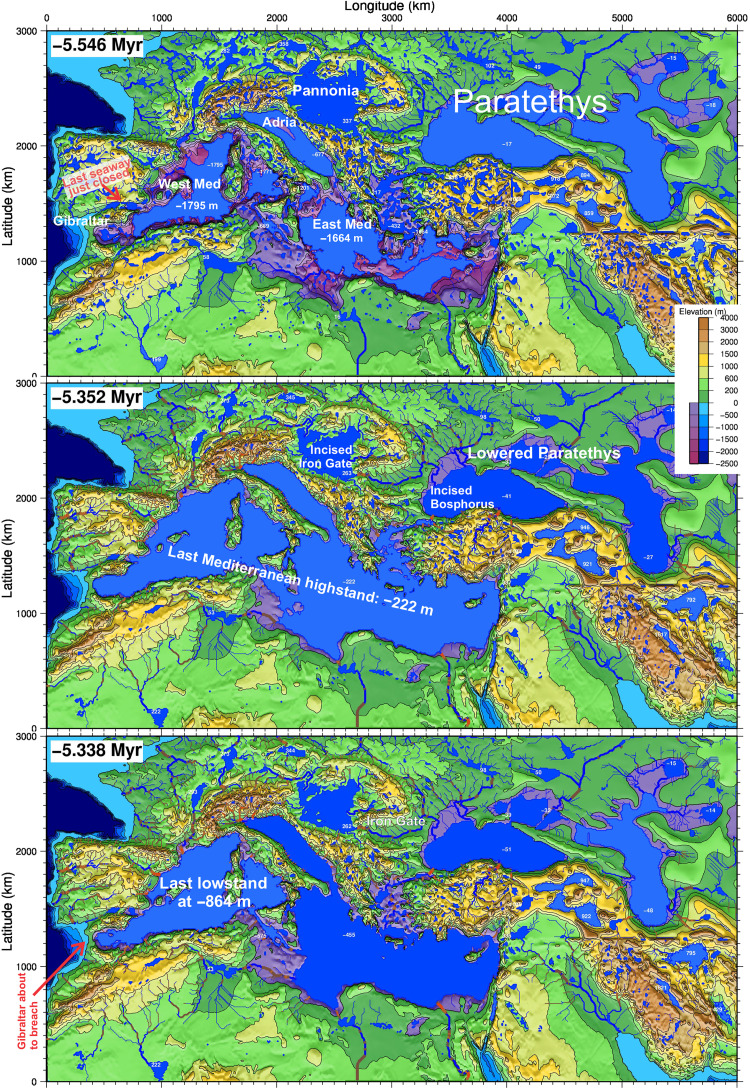
Results for the reference LEM M0. Three stages of the drainage network and the topography at −5.546 Myr (first dry precessional lowstand after full isolation), −5.352 Myr (last wet highstand and insolation maximum), and −5.338 Myr (last lowstand before Pliocene). Light and dark blue indicate endorheic and exorheic lakes, respectively. Red river segments indicate obtained erosion from 40 m to more than 600 m of incision (see Fig. 6). The shrinking of the Paratethys lakes is due to erosion along their outlets at the Bosphorus, the Aegean Sea, and the Iron Gate gorges. White numbers indicate lake levels. The overall level rise of the Mediterranean since the first until the last precessional lowstands is due to the excess water that is no longer evaporated in the Paratethys. The paleogeographic reconstruction is approximative, and the specific results for individual basins and rivers must be considered with caution. The full model evolution is available as movie S1.

Each of our model runs starts from the same initial paleogeographic reconstruction, corresponding to 5.55 Myr ago, age of the first evaporative drawdown (*30*, *38*). This topographic reconstruction (Fig. 1) is based on earlier studies from the Pannonian Basin (*39*), Caspian and Black seas (*40*), Chad-Eosahabi river in present Libya (*41*), West Mediterranean (*22*), and Alborán (*27*). In addition, we restore the counterclockwise rotation of Africa relative to Eurasia, which has shrunk the East Mediterranean by up to 105 km in the N-S direction since the Latest Miocene. On this initial surface, water runoff (hereafter referred to as “precipitation” *P* and expressed in millimeters of rain per year; Table 1) is assumed to follow a linear dependence with elevation, longitude, and latitude based on today’s precipitation patterns. Precipitation is also assumed proportional to average insolation at 65 N following Milankovitch’s cyclicity (*42*), implying oscillations in precipitation of up to 12% (*41*), with maximum precipitation values occurring during insolation maxima. Each model run covers the entire Lago-Mare (stage 3), from *t =* −5.55 Myr until the last dry cycle before the Pliocene (−5.338 Myr).

**Fig. 2.**
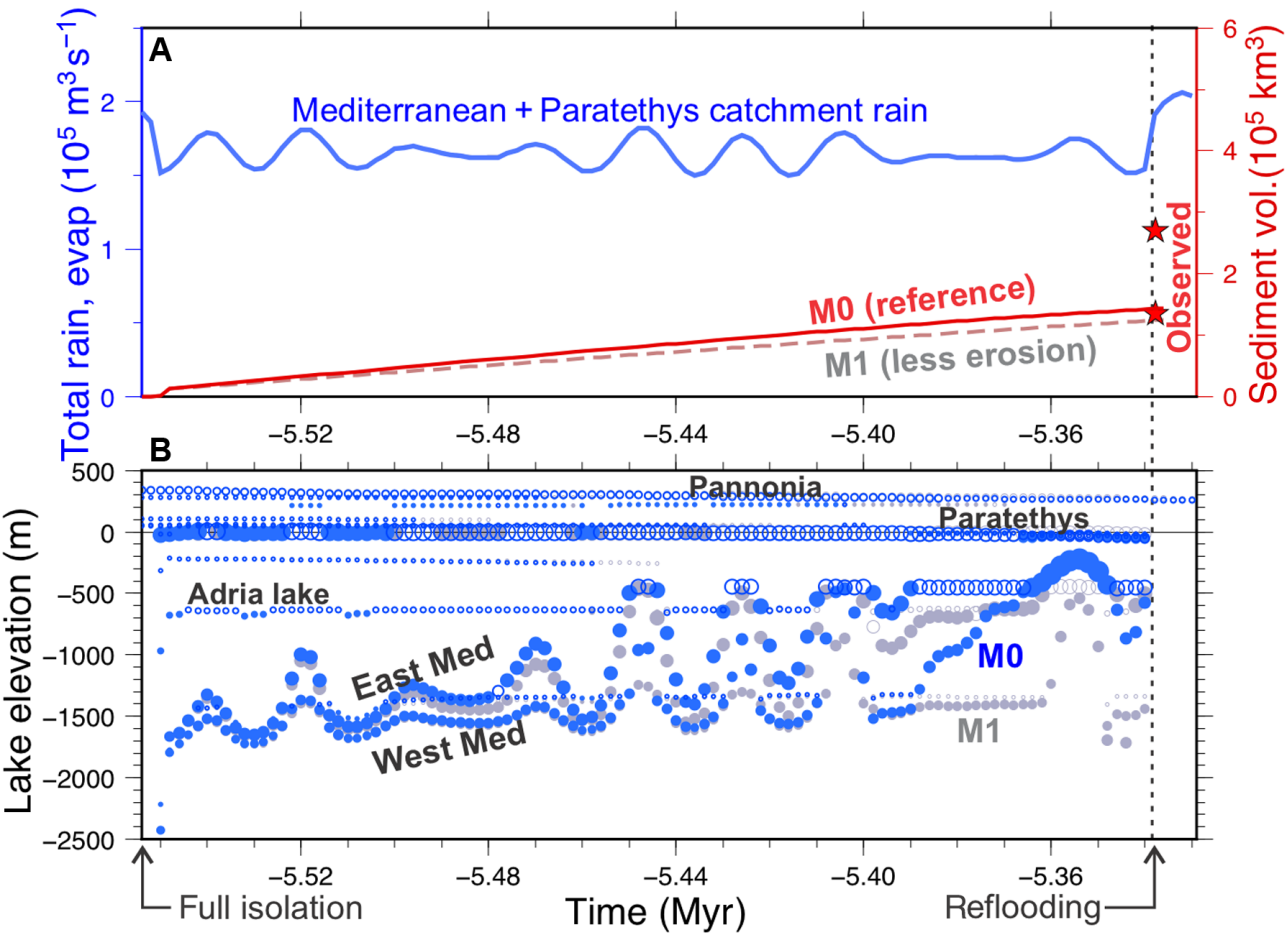
Lake elevation through time and its sensitivity to erodibility and climate. (**A**) Evolution of the total precipitation (blue) over the entire model domain reflecting the orbitally induced cyclicity and the evolution of the sediment volume accumulated in the Mediterranean (red line), for the reference model M0 (Table 1). Results for the setup M1 (20% less erodibility *k*_b_) are shown in gray colors). Red stars indicate the estimated range of MSC detrital sediment (*43*). (**B**) Evolution of lake elevation for M0 (blue) and M1 (gray). Circle size is proportional to lake area; open circles indicate open lakes, and filled circles indicate endorheic lakes. Main lakes initially oscillate at very low elevations from −1800 to −1300 m, whereas during the last stages, they receive more water from the Paratethys, nearly reaching the ocean level (*z* > −100 m) during highstands. In contrast, M1 (gray) predicts less erosion, less water capture from Paratethys, and lower lakes levels in the Mediterranean.

Because of uncertainty in many of the parameters involved, LEMs have limited capability to correctly estimate erosion rates unless independent constraints on the erosion/sedimentation balance are used to calibrate the model. For this purpose, we use the MSC sediment budget based on a Mediterranean-wide compilation of seismic stratigraphy (*29*, *43*, *44*), which constrains the volume of clastic sediment accumulated during the MSC at 266,000 to 372,400 km^3^. Recent analyses of cuttings from the Aphrodite well in the Levant Basin (*6*) show that clastic content in the halite unit is substantially lower than assumed in that compilation (*43*). For this reason, we discard the higher value and consider instead 50% lower sediment volume for comparison with the model results (red stars in Fig. 2). Well-constrained local examples of this sediment budget include the Handere (*29*) and Nahr Menashe (*45*) formations offshore eastern Anatolia at 8300 and 4150 km^3^, respectively. For reference, these volumes roughly match the pre-dam Nile sediment load of 1.24 × 10^11^ kg year^−1^ extrapolated to the 210–thousand year (kyr) duration of stage 3.

The precipitation parameters are chosen (Table 1) to fit the average water deficit of the Mediterranean during the Messinian. This has been estimated to be between −68,100 and −57,000 m^3^ s^−1^ (*41*), somewhat wetter (less negative) than today’s budget (*24*), estimated between −75,000 and −50,000 m^3^ s^−1^ (*46*). We then add an oscillation in precipitation due to orbital precession (*41*), assuming that *P* is proportional to the average summer insolation at 65 N. This causes time changes in runoff of up to 12%. The resulting water discharge at each cell of the topographic model allows us to perform forward LEM modeling of erosion to fit the MSC sedimentary budget by modifying the bedrock and sediment erodibility. We choose a reference model setup M0 that best fits the sedimentary budget and the water balance. Ultimately, what validates this reference setup M0 is that it satisfies both the hydrological deficit of the Mediterranean and the 145,000 km^3^ of sediment accumulated in the Mediterranean for the 210 kyr of stage 3, both within the uncertainty range of measurements. Other parameter values for M0 are listed in Table 1.

### Results for mechanically plausible MSC scenarios

**Fig. 3.**
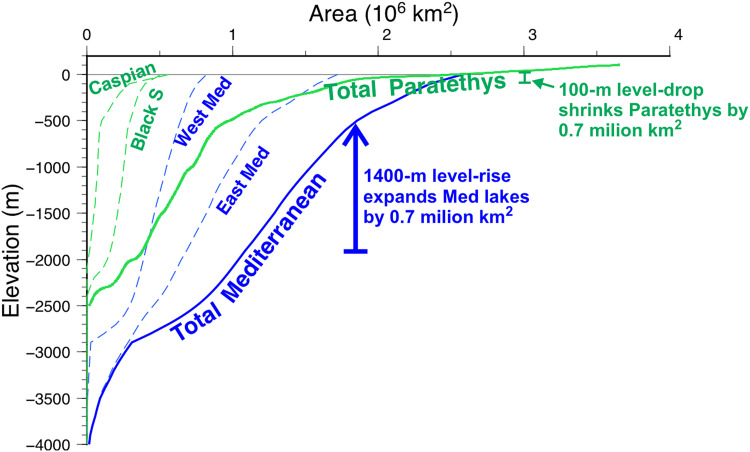
The hypsometry of the Mediterranean and Paratethyan basins provides a simplified version of the model mechanics. A 100-m lowering of the Paratethys lake system from 10 to −90 m (as in M0 at *t* = −5.38 Myr) implies a reduction by 0.7 million km^2^ in lake evaporation area. This loss in evaporation area is compensated by identical expansion of the terminal Mediterranean lakes, implying a rise in the Mediterranean level from −1900 to −550 m. This is how the hypsometry of the Paratethyan and Mediterranean domains amplifies the climate- and erosion-related variations in the hydrological balance in the former as large sea level changes in the latter.

The topographic and drainage evolution resulting from the reference model setup M0 (Table 1) is shown in Figs. 1 and 2. The first evaporitic drawdown peaks during the insolation minimum at *t* = −5.546 Myr, leading to a substantial desiccation of the Mediterranean, with the western lake level at −1795 m and the eastern basin at −1664 m (Fig. 2) (elevations relative to present sea level). The large lakes forming in the Pannonian Basin (+337 m above present sea level) and Paratethys (−17 m) initially contribute little excess water to the lakes formed in the Mediterranean. The Pannonian Lake overtops toward the Paratethys lake through the threshold near the present Iron Gate (Romania). The Paratethys starts as an endorheic lake, but as soon as the first wet precession maximum arrives, at *t =* −5.54 Myr (*42*), it overflows (Fig. 2B) toward the E Mediterranean through the Bosphorus Strait and the Aegean Sea. These exact timing, drainage, and lake elevation results are very sensitive to the elevation adopted for the topographic sills, which is poorly constrained. However, what is relevant to the present study is that 210 kyr later, these two outlets have become eroded by 74 and 50 m (see red lines in Fig. 1), leaving the respective lakes at elevations of +262 and −51 m. Consequently, the Pannonian and the Paratethys lakes see their average areas reduced by 635,000 km^2^. To keep the balance with the water sources, this lake shrinking is compensated by an identical area expansion of the Mediterranean lakes. This water transfer is performed in the model by an ever-growing river that forms along the Aegean seafloor from NE to S. The excess water that is no longer evaporated in the Paratethys (24,100 m^3^ s^−1^) is delivered to the Mediterranean, which rises up to −222 m during the last wet phase before the Miocene/Pliocene boundary (Fig. 1; *t* = −5.352 Myr). Each rise of the Mediterranean level substantially amplifies the drop of the Paratethys because the latter starts at a level close to zero, where its hypsometry curve is very flat (Fig. 3), whereas the Mediterranean Sea middle depths are dominated by steep slopes, owing to the tectonic origin of this basin.

The main limitation to interpreting these results comes from the uncertainties intrinsic to the paleogeographic reconstruction. For example, the depth of the threshold between the E and the W Mediterranean (the unknown paleodepth of the Sicily Sill) is arbitrarily set at −440 m for M0. This determines the diachronic behavior of both sides of the sea: A deeper value for this sill results in a less diachronous evolution. However, this limitation does not compromise the central focus of this study: the gradual lake-level rise in the Mediterranean driven by drainage integration.

**Fig. 4.**
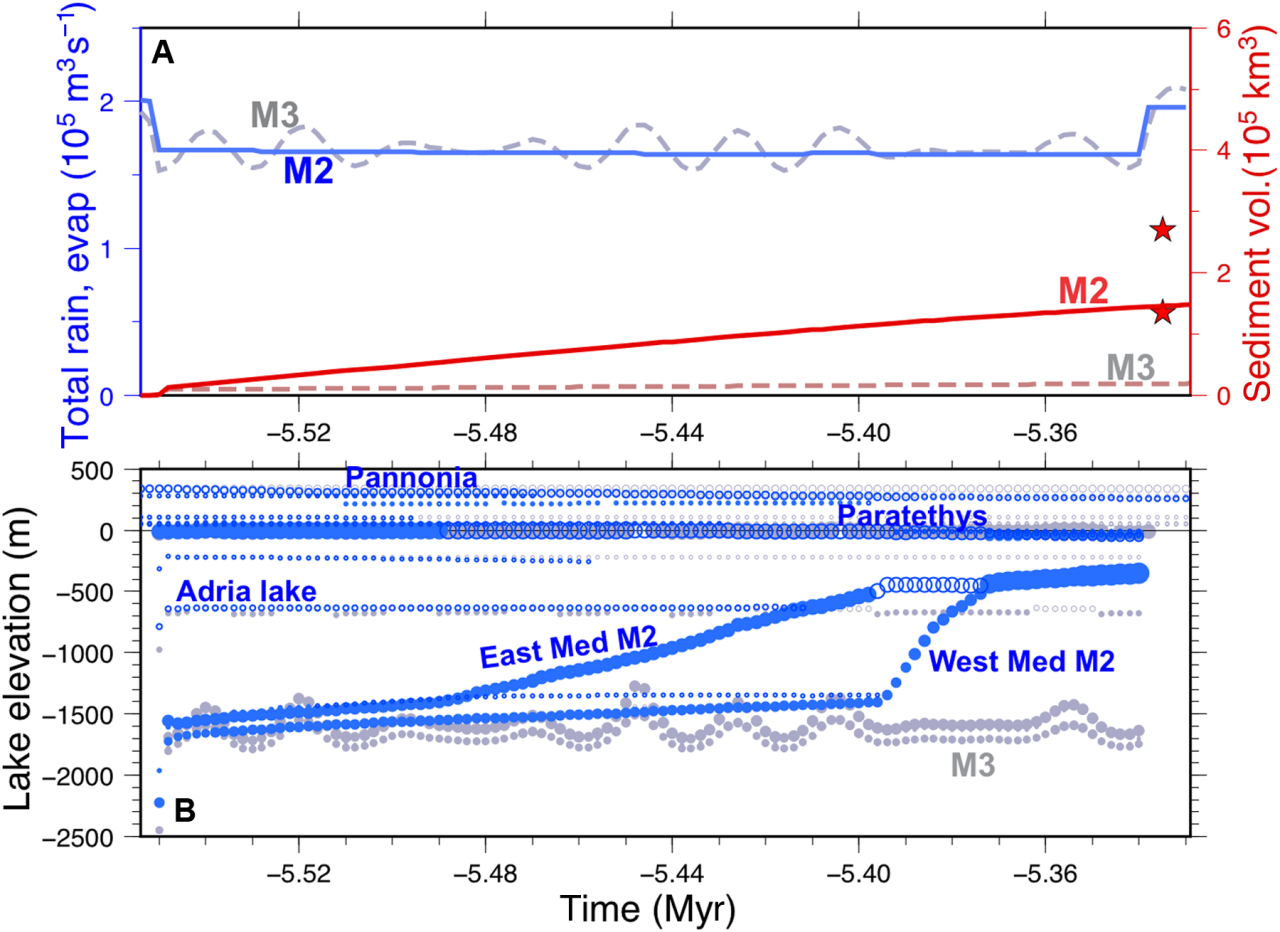
Significance of spillway erosion and climate oscillations on the evolution of the lakes’ elevation. (**A**) Evolution of the total precipitation (blue) and accumulated sediment volume (red). (**B**) Lake elevation evolution for two hypothetical scenarios identical to the reference M0, except M2 (blue) disregards orbital climate changes and M3 (gray) disregards lake outlet erosion. Other legend as in Fig. 2. Outlet erosion is the process that allows the Mediterranean lakes to rise from deep shallow lakes to a nearly full basin.

We identify lake outlet erosion as the main mechanism controlling this gradual kilometric level rise of the Mediterranean Sea. Figure 2 compares the results obtained for the reference model M0 with an identical setting adopting 20% lower erodibility (harder rock), labeled M1 and showing the substantial delay induced in the rise of the Mediterranean lake levels. To better isolate the effects of climate and erosion, Fig. 4 shows the results for model M2 (identical to M0 but disregarding the precessional climate cyclicity) and M3 (as M0 but disregarding erosion). The results show that insolation cyclicity only accounts for lake level changes of up to 600 m, whereas erosion causes an overall lake level rise of more than 1300 m.

Apart from rock erodibility, the main parameter determining the evolution of the lake levels is the rainfall pattern (runoff). The results obtained for 10% lower precipitation (M5) are compared to those of M0 in fig. S2. The greater accumulated sediment volume is attributed to the drier climate, which results in lower lake levels and longer exposure of the seafloor to fluvial incision.

**Fig. 5.**
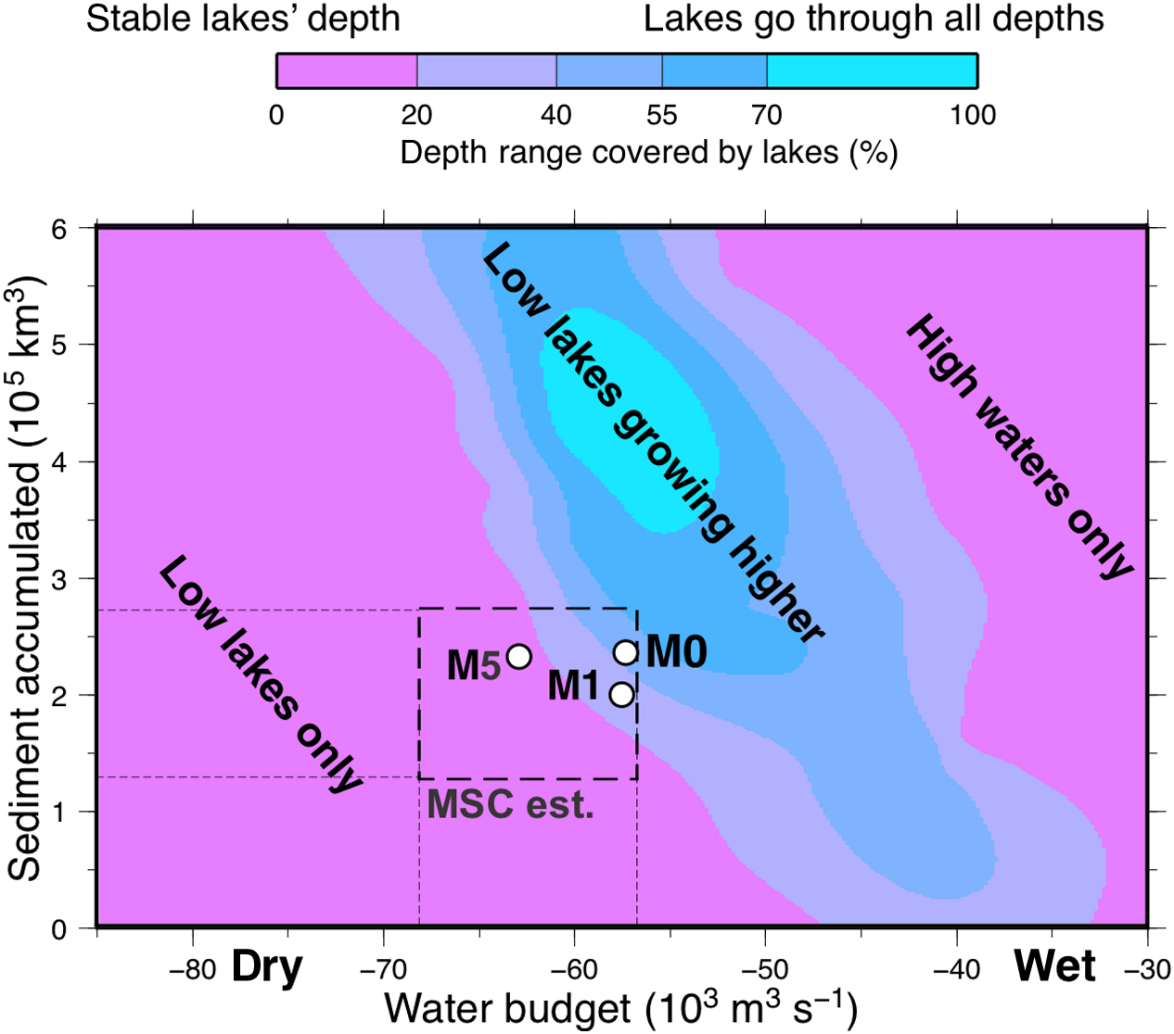
Lake elevation sensitivity to the hydrological and sedimentary balance, model sensitivity test. The color shade shows the percentage of depths covered by Lago-Mare lakes as a function of the adopted value for the water budget and for the accumulated MSC sediment. Dashed box indicates the range estimated for the MSC. The circles locate results from specific model runs. For dry climate (low *P*) and low erosion/sedimentation (low *k*_b_), the Mediterranean remains mostly dry, at low levels that do not increase over time (green shade). Inversely, wet climate and high erodibility quickly lead to a full Mediterranean basin incompatible with the kilometer-deep erosion marks (green). Only some combinations of climate and erosion (blue strip) lead to a similar presence of both low and high lakes during the model’s evolution, compatible with the sedimentological and seismic stratigraphy data. That strip overlaps with the range of sediment volume and climate estimated for the MSC (dashed box) near the model run adopted as reference (M0), explaining the puzzlingly evidence for both a high- and a low-level Mediterranean during the Lago-Mare (stage 3).

To constrain how sensitive this prediction of a rising Mediterranean level is to the adopted parameters, we explore the field of combinations of climate and erodibility that satisfy both the hydrological and the sedimentary budgets (Fig. 5). For this parameterization, we simplified the climate model using a harmonic oscillation of constant amplitude for precipitation, rather than the insolation curve, so the sediment volumes obtained for the M0, M1, and M5 setups do not match exactly those in Figs. 2 and 4. The color shade in Fig. 5 shows what percentage of the Mediterranean depth range is covered by the model lakes at some point during the model evolution. A remarkable result is that the observed MSC sediment volume and hydrological budget can be reproduced when the levels of the Mediterranean lakes cover most of the depth range during the time span of the Lago-Mare stage.

**Fig. 6.**
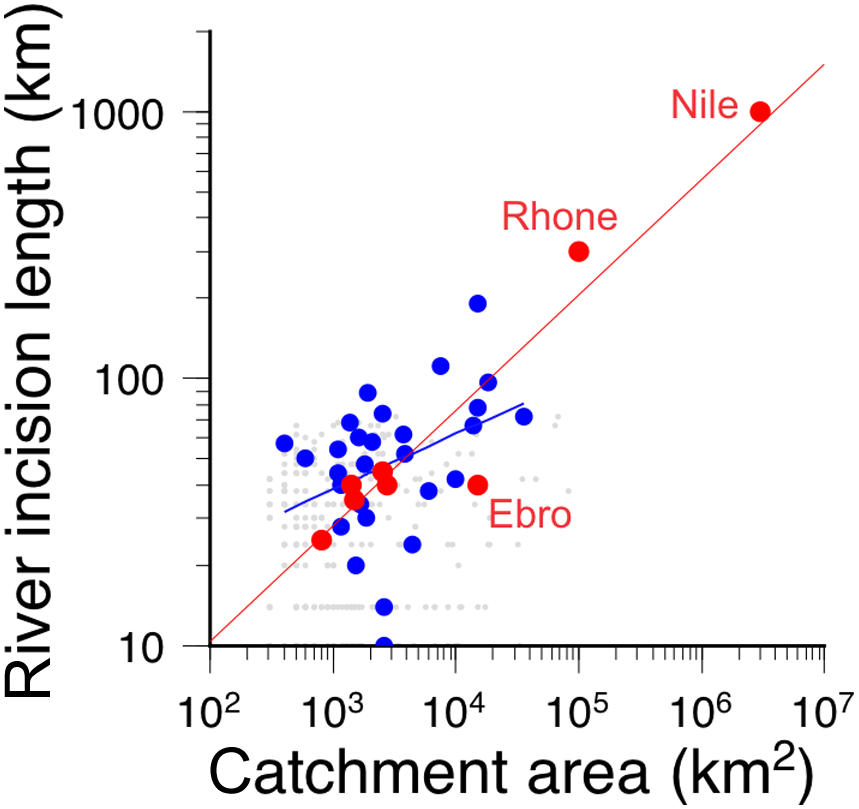
Cumulative river incision in M0 (blue) compared to observations along the Mediterranean margins (red), as a function of drainage area. Although the river incision lengths caused by the MSC drawdown are subject to interpretation, the resemblance between the model and the observations provide independent validation of the erosion calculated in the model. The length *L* to drainage area *A* relationship obtained in (*47*) is *L* = 1.3 *A*^0.45^, steeper than the model’s *L* = 3.5 *A*^0.31^. The mismatch is mostly recovered if the model duration is duplicated, but given the difficulties inherent in measuring the length of Messinian valleys and their ancient catchment area, we opt to prioritize the fit of MSC sediment volumes rather than these data. Observations after (*47*); the Ebro value has been corrected for a longer found incision channel (*65*) and for its drier climate and lower discharge relative to the Nile and the Rhone.

Last, to independently validate the speed at which the TISC LEM performs the fluvial incision, in Fig. 6, we compare the observed length-to-drainage area distribution of reported fluvial incisions formerly attributed to the MSC (*47*). While M0 captures the overall trend of longer incision propagation at larger catchments, the documented lengths of incised valleys (red dots in Fig. 6) require larger erodibility values than the ones used above. We attribute the difference to the interpretation involved in estimating the length of those incised valleys and favor the sedimentary budget as a more reliable and less interpretative dataset to define the reference model M0.

## DISCUSSION

### How to explain all the levels of the Mediterranean

**Fig. 7.**
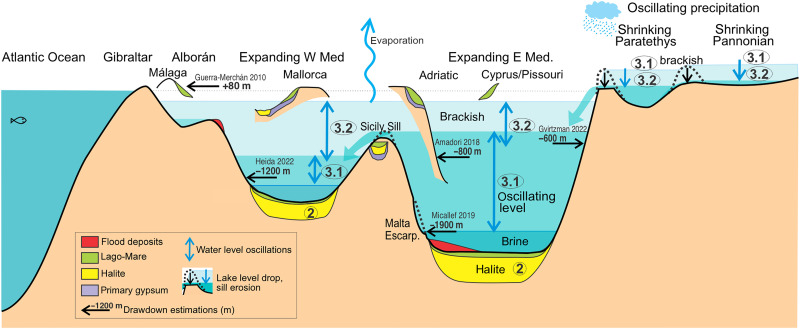
Summary of conflicting evidence on the Mediterranean Sea levels during MSC stage 3 and conceptual model for a gradual rise of sea level oscillations due to erosion along lake outlets. The initial sea level drop during stage 2 leaves a nearly empty basin, which is then modulated by precession during stage 3.1. As the higher Paratethyan lakes shrink in area due to outlet erosion, the water no longer evaporated there is delivered to the Mediterranean. That lake area reduction must be compensated by an equal lake area gain in the Mediterranean lakes, since the same amount of precipitation must be evaporated in the endorheic system. This causes a gradual rise during stage 3, explaining the presence of nearly desiccated basins during the first dry precession periods, a nearly full Mediterranean during the last wet periods, and yet a low-level Mediterranean during the last dry precessional cycle, allowing for a rapid (cataclysmic) reflooding to restore normal marine conditions at the Miocene/Pliocene boundary. Not to scale.

**Fig. 8.**
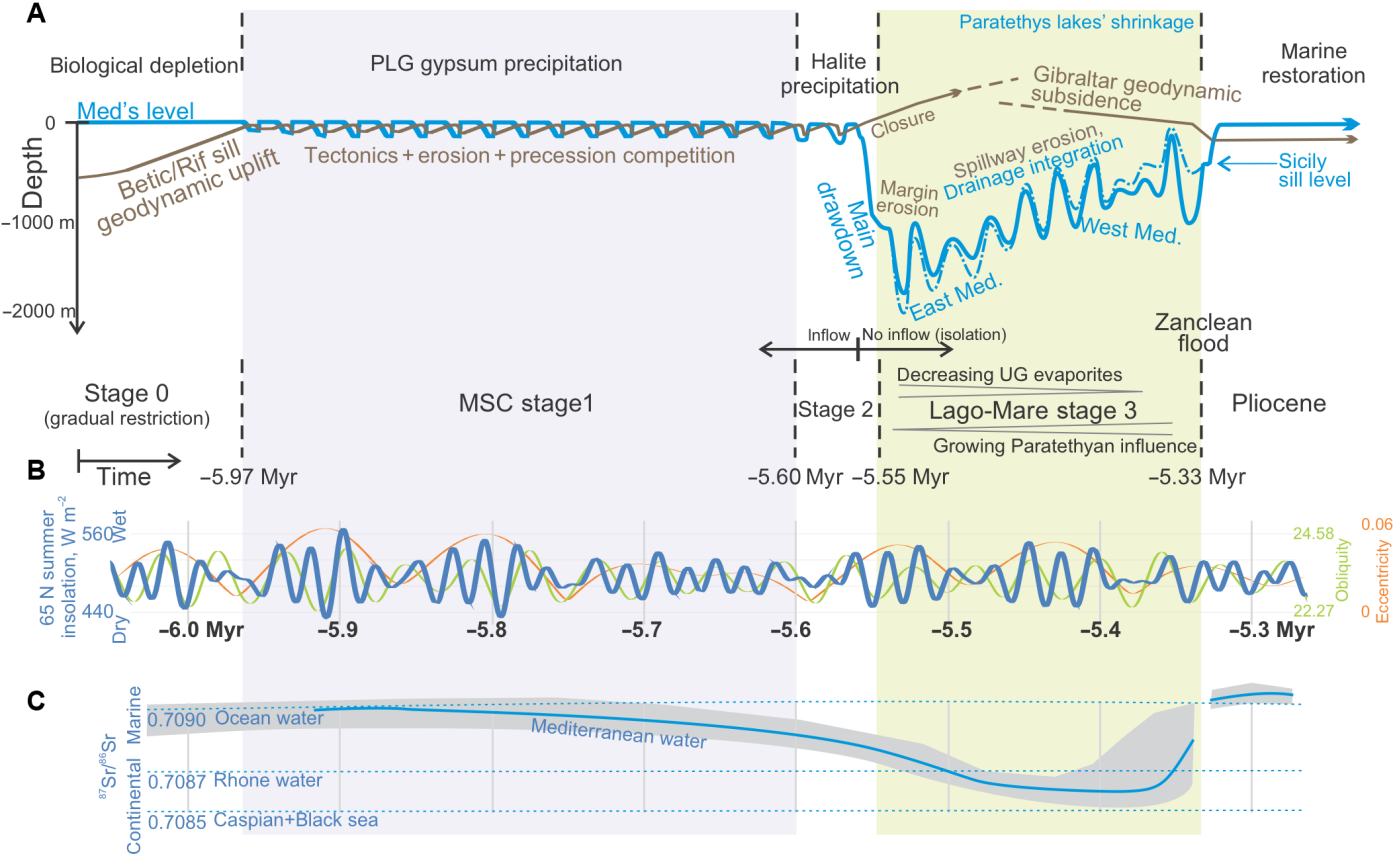
Mechanistic model for the evolution of the MSC. Total MSC duration: 630 kyr, 29 precession cycles. (**A**) Interpretations linked to tectonic and erosive processes (brown) and hydrological and climatic processes (blue). (**B**) Insolation at 65 N (*42*) and orbital eccentricity and obliquity. (**C**) Strontium isotopic evolution of Mediterranean basins compared to the ocean and to other water sources. Stage 1 (represented by 16 gypsum cycles) is a long period of competition between uplift and seaway erosion in the last remaining Betic seaway (*1*). Stage 2 (3 cycles) encompasses the bulk of halite precipitation, mostly during the largest evaporitic drawdown of its water level: The most recent salt balance (*43*) brings the amount of salt close to what would cause the evaporation of a saturated brine the size of the Mediterranean. Stage 3 (10 cycles): Our results indicate that the transition from stages 3.1 (upper evaporites) and 3.2 (lower salinity) are the natural outcome from the retrogressive erosional wave propagation into the Paratethys realm after the first desiccation during stage 2. The implied increasing discharge of Paratethyan brackish water to the Mediterranean explains the gradual continentalization of the strontium isotopic signal (*9*). This suggests, together with numerical models of the erosion produced by the inflowing water (*1*), that the drawdown during stages 2 and 3 occurred fully disconnected from the ocean (no Atlantic inflow). Restoration of normal marine conditions is triggered by slab pull–driven subsidence of the westernmost Betic Cordillera, leading to the formation of the Strait of Gibraltar and to the Zanclean flood. PLG, primary lower gypsum; UG, upper gypsum.

Oscillations in the hydrological budget due to orbital cyclicity caused sea level oscillations in the Mediterranean of up to 600 m in amplitude (Fig. 4) (*41*). However, these oscillations alone cannot explain the shallow ostracod fauna and facies found in stage 3 upper evaporites that were cored more than 2 km below present sea level in the abyssal plains (*10*) and the brackish water deposits formed at around today’s ocean level. This is best shown by the model run M3, which disregards the effects of spillway erosion, demonstrating that climatic oscillations alone can neither explain the whole depth range of MSC sea level markers. Therefore, we conclude that the missing mechanism complementing cyclicity is the gradual capture of the surrounding basins’ (Figs. 7 and 8). This retrogressive erosion and drainage integration reduces water evaporation in the surrounding continental areas (mainly the Paratethys lake) and leads to a progressively higher Mediterranean water level. The volume of clastic sediment accumulated in the Mediterranean during the MSC constrains this model of spillway erosion during the Lago-Mare (stage 3).

The relatively flat hypsometry of the Paratethys basins near the present sea level (Fig. 3) meant that modest amounts of erosion along its outlet led to a significant reduction in lake area, resulting in a large increase in excess water delivered to the Mediterranean (Fig. 7). This process might have been facilitated by the preexistence of an outlet from the Paratethys to the Mediterranean before the MSC (*14*). Supporting this idea, the arrival of the Paratethyan immigrant fauna began as soon as the drawdown was accomplished, with *Loxoconcha muelleri* being documented only 20 cm below an ash layer dated at 5.532 Myr ago (*48*, *49*).

The fall of the Paratethys lake level might be greater than obtained in M0, as it is vaguely estimated at about a hundred meters during the MSC (*14*, *16*, *17*) based on sedimentation hiatuses. Geophysical data yield even larger drop estimations (*18*). Our model suggests an erosional mechanism for this level drop (model M0 reaches a drop of −57 m; Fig. 1) and demonstrates that this fall in the Paratethys level may have been the cause, rather than the consequence, for the evolution of the Lago-Mare (stage 3). A fall of 100 m due to spillway erosion would imply a shrinking of the Paratethys lake evaporation area by about 700,000 km^2^ (Fig. 3), resulting in an extra water supply to the Mediterranean of ~26,000 m^3^ s^−1^ and its level rise by about 1400 m.

The proposed retrogressive erosional wave propagating into the continent can explain several characteristics of the changing Mediterranean sea level during stage 3. First, the fact that previous estimations of the water level drawdown, based on erosional and depositional markers (*27*), suggested a wide range of depths, from close to zero (present sea level) down to −2200 m (Fig. 7) (*29*). For example, in light of the results, the puzzling high-elevation Lago-Mare deposits close to present sea level in (from W to E along the southern coast of Iberia) Málaga Basin (*31*), Sorbas Basin (*32*), Vera Basin (*13*), Mallorca Basin (*50*), and many other outcrops (*23*) can be seen as the consequence of the latest highstands of stage 3. In contrast, the nearly empty basin scenario suggested by the shallow lacustrine deposits found in boreholes at the deepest basins (*10*) could have taken place during the earlier dry precessional periods (insolation minima; Fig. 8). These shallow basins in the deeper parts of the empty Mediterranean during the earlier dry precession periods of stage 3 (5.53 insolation low) gradually turned into a lower salinity and nearly filled basin of more Paratethyan-sourced water during the late wet-climate highstands, toward the end of stage 3. As excess water from the Paratethys increased, the Mediterranean oscillations gradually reached higher levels, and the Mediterranean water became more diluted, allowing for the sedimentation of the Lago-Mare during highstands and its eventual erosion during lowstands, as seen in outcrops such as the Almanzora Caves (SE Spain) or the Málaga Basin (N coast of the Alborán Sea) (*31*, *51*). Note that the model does not impose any interbasinal connectivity changes other than by the erosion of lake outlets, so it is this mechanism that causes the transition to higher water levels during stage 3 (Fig. 8). The predicted gradual capture of Paratethyan waters also provides an explanation for the minimum strontium isotopic ratio that occurs late during the Lago-Mare (Fig. 8), since it is the Paratethys realm that is characterized by the lowest ratios.

The spillway erosion mechanism also provides a simple explanation for the presence of halite patches at mid depths, most prominently in the Central Mallorca Depression (CMD) in the Balearic Promontory (*22*, *52*), in contrast with the absence of halite elsewhere at the margins of the Mediterranean. This absence has previously been related to water stratification preventing salt precipitation in shallow waters. However, this argument conflicts with the salt precipitation observed in the modern Dead Sea, prevailing at depths of just a few tens of meters. The extreme current Dead Sea drawdown of about a meter per year allows the warm summer water to dissolve most of the halite precipitated during winter near the shore (*53*). Taking this analog to the MSC, it seems plausible that halite originally precipitated over most of the Mediterranean and then the sea level oscillations provided 20 opportunities to wash out the salt along the moving coastline (10 cycles of 21,700 summers each). That is, the sea level cyclicity provides more effectiveness to the marginal erosion mechanism earlier proposed to explain the absence of salt at mid-depths along the margins (*52*). Only in local bathymetric minima such as the CMD or Caltanissetta (Sicily), this salt remained shielded from this coastal washing and preserved until nowadays (*22*).

The proposed mechanism for Mediterranean Sea level rise also provides an explanation as to how the higher Lago-Mare deposits can be compatible with the drawdown of at least some hundreds of meters required for the Zanclean megaflood to happen (*11*). The end of the MSC and the transition to normal marine conditions at the Miocene/Pliocene boundary was initially seen as a process taking up to 10,000 years (*54*), but increasing evidence suggests a much faster refill of cataclysmic nature, lasting less than a few years and involving an unprecedented water discharge (*11*, *12*, *55*, *56*). However, the seeming incompatibility of this event with the nearly full Mediterranean implied by the highest Lago-Mare deposits has been puzzling. Our model suggests that the highest Lago-Mare sediment could have been deposited during the last precessional highstands before the last drawdown that facilitated the breaching of the Gibraltar Sill (*11*). An issue raised by the results, however, is that the last drawdown of the Eastern Mediterranean is predicted to be moderate (less than 1 km; Fig. 2), conflicting with the idea that the Zanclean flood was responsible for the morphology of the 2-km-deep Noto Canyon offshore Sicily (Fig. 6) (*55*).

Last, our model poses two testable predictions for future research. First, the thresholds between subbasins should have been detectably eroded by tens or hundreds of meters as basins repeatedly spilled into one another. This may help identify the sills separating the Adriatic Sea, the Western Mediterranean and the Aegean Sea from the Eastern Mediterranean in the seismic stratigraphic record (*57*). Second, basins should have become overtopped earlier in the East (Aegean, Levant, and Ionian seas) than the western basins (Adriatic, West Mediterranean, and Alborán basins), in an overall E to W water refill from Paratethys to Alborán. The pathway of the inflow of Paratethyan water into the Mediterranean Sea is itself also to be localized.

### Toward a comprehensive model for salt giant evolution

Recent modeling of the chlorine isotopic content in MSC halite (*6*) adds to previous models for the closure of the Atlantic-Mediterranean connection (*1*, *58*, *59*) to conclude that halite may have started to precipitate at a sea level similar to the present ocean’s and ended after full isolation during the main initial kilometric drawdown. Halite may have precipitated with a markedly distinct timing in the E and W basins. From this perspective, halite precipitation might not be the best criterion to define a separate stage 2. Instead, the first halite could be ascribed to a stage 1 defined by a full Mediterranean, while the late halite can be seen as belonging to the Lago-Mare (stage 3), characterized by a nearly complete disconnection from the ocean and a large, oscillating drawdown. Similarly, the model predicts that the transition to fresher conditions during the Lago-Mare is the natural result of drainage integration. This suggests that substages 3.1 and 3.2 resulted from the gradual capture of peri-Mediterranean waters due to the erosion of outlets. The MSC may thus be described by just two distinct stages: a first one at normal sea level with decreasing outflow and inflow caused by the tectonic restriction of the gateway (*1*); and a second stage of canceled oceanic inflow, full isolation, and large evaporitic drawdown modulated by the mechanisms modeled in this study.

Tens of giant salt accumulations similar to the MSC have formed over the history of Earth, but their impact on ecosystems and on the global ocean geochemistry remains underexamined. The recent discovery that at most 11% of the Mediterranean marine species survived the MSC (*4*) demands a better understanding of what were the possible refugees and what was the drainage and landscape evolution during these events. Our results suggest that the sea level drop was very dynamic and heterogeneous, facilitating a succession of diverse environments including river deltas and separate subbasins with diachronous salinity changes and stratification. This may have provided the opportunity for some marine species to survive throughout the MSC. Other salt giants involving a full disconnection from the ocean should have followed a similar evolution. Transposing this model to the evolution of older salt giants will enhance our understanding of the role these abrupt episodes play in system Earth evolution.

## MATERIALS AND METHODS

The LEM algorithms for drainage and erosion calculation are explained in more detail in (*37*). Essential to our results is the algorithm to determine the drainage network accounting for lake evaporation and endorheism. It starts by determining the fluvial network. Each river cell will drain to the lowest of its eight neighbors in the rectangular grid. This steepest slope network is completed with the identification of lakes in local topographic minima, initially considered exorheic and overspilling along the lowest surrounding sill or threshold. Then, this network is followed again top-down adding rain precipitation *P* (in millimeters per year) to each cell and transferring water discharge (in cubic meters per second) along the river paths and lake sills. TISC assumes steady flow, i.e., water flow transitory stages such as floods are not accounted for, and water inputs from precipitation are perfectly and instantaneously matched by lake evaporation and water loses through the boundaries. Evapotranspiration loses are subtracted from rivers and lake cells and the lakes’ area is reduced if they become endorheic (i.e., if they receive less water than they evaporate at the surface). This lake shrinkage is performed by removing the highest grid cells from the lakes until their evaporation matches water inputs. This lake surface adjustment algorithm is far from simple. For example, when removing lake cells, lakes can become split in several smaller lakes. Depending on the new water budget of these separate lakes, some of them may become exorheic, and the removed cell may become a spillway prone to be eroded by water discharge following Eq. 1, rather than accumulating lake sedimentation. This process makes TISC’s algorithm more costly in calculation time compared to other LEMs in the geoscience community. For reference, model M0 needs about 8 days to run in a single Apple M2 processor.

Once the water discharge along the network is computed, the algorithm calculates erosion making use of a version of the stream power approach. We couple the Manning’s formula for water flow and an empirical relationship for channel width with the erosion rate *e* written as a function of basal shear stress, the following stream power law dependence on water discharge *Q*_w_ and slope *S* is obtained (*37*) for the rate of erosion/sedimentation *e*

$$e=-\frac{\mathrm{dz}}{\mathrm{dx}}=\frac{q_{\text{eq}}-q_{s}}{q_{\text{eq}}}K_{e}\;Q_{w}^{m}\;S^{n}$$ (1)

where *e* is the incision rate along the river channel of slope *S*, water discharge *Q*_w_, and sediment load *q*_s_. When the river carries more sediment than the equilibrium transport capacity *q*_eq_, then Eq. 1 turns negative, implying sedimentation. *q*_eq_ is calculated as proportional to the water flow energy

$$q_{\text{eq}}=K_{t}\;Q_{w}\;S$$ (2)

A value of *K*_t_ = 1000 kg m^−3^ is adopted from (*36*). As for the efficient erodibility *K*_e_, it can be calculated as

$$K_{e}=k_{b}\;{\left(\rho \;g\right)}^{a}{\left(\frac{n_{r}}{k_{w}}\right)}^{3a/5}$$ (3)

$$m=\frac{3\;a\;\left(1-a_{w}\right)}{5};n=\frac{7\;a}{10}$$ (4)

where *k*_b_ represents erodibility that relates basal flow shear stress τ to erosion in $e=k_{b}$τ, *n*_r_ is the roughness coefficient, ρ is the density of water, *g* is the acceleration of gravity, and *k*_w_ and *a*_w_ are the constants of the power law for channel width as a function of water discharge [details in (*37*)]. We adopt values of *n* = 0.05, *a* = 1.5 (*60*), *a*_w_ = 0.5, and *k*_w_ = 1.2 (*61*–*63*). The main uncertainty in the calculated erosion rates *e* comes from the erodibility, which, for long-term fluvial incision, can span over more than four orders of magnitude (*64*). For this reason, pinning the volume of rock eroded and sediment transported is crucial for obtaining reliable drainage-change predictions. The abrasion effect of sediment tools on erosion is not considered, both for the sake of simplicity and because the key locations that will be found responsible for the model evolution are lake outlets, characterized by clear water with a small amount of sediment load.

To keep the model to a minimum number of unconstrained parameters, runoff is considered linearly proportional to latitude, longitude, and elevation, using proportionality constant constrained from today precipitation distribution in the model area. Lake evaporation rate is also considered constant for simplicity, disregarding changes introduced by salinity, temperature, wind, or elevation. The effect of orbitally controlled changes in precipitation is accounted for by using Laskar’s curve for insolation (*42*) and adopting a proportionality between insolation and *P* matching the results by (*2*), inducing variations if *P* of up to ±12%.

Rather than making the model less reproducible by introducing further complexity with spatial and temporal variations of parameters that are poorly and patchily constrained for the late Miocene, we focus the modeling on constraining the timescales of propagation of the erosional wave caused by the sea level drop, our starting hypothesis. The algorithm is explained in more detail in ref. (*37*) and in the code itself, publicly available at https://doi.org/10.5061/dryad.r2280gbpf.
