# Supplementary material for: Kilometric sea level changes during the Messinian salinity crisis caused by river erosion and climate
Source: Sci Adv. 2025 Jul 11;11(28):eads9752. doi: 10.1126/sciadv.ads9752 (PMC12248288; doi:10.1126/sciadv.ads9752)
Supplement: Supplementary file 1 — Figs. S1 and S2 Legends for movies S1 to S3 [file sciadv.ads9752_sm.pdf]

Science Advances
AAAS

Supplementary Materials for

Kilometric sea level changes during the Messinian salinity crisis caused by river erosion and climate

Daniel García-Castellanos *et al.*

Corresponding author: Daniel García-Castellanos, d.g.c@csic.es

Sci. Adv. **11**, eads9752 (2025)
DOI: 10.1126/sciadv.ads9752

The PDF file includes:

Figs. S1 and S2
Legends for movies S1 to S3

Other Supplementary Material for this manuscript includes the following:

Movies S1 to S3

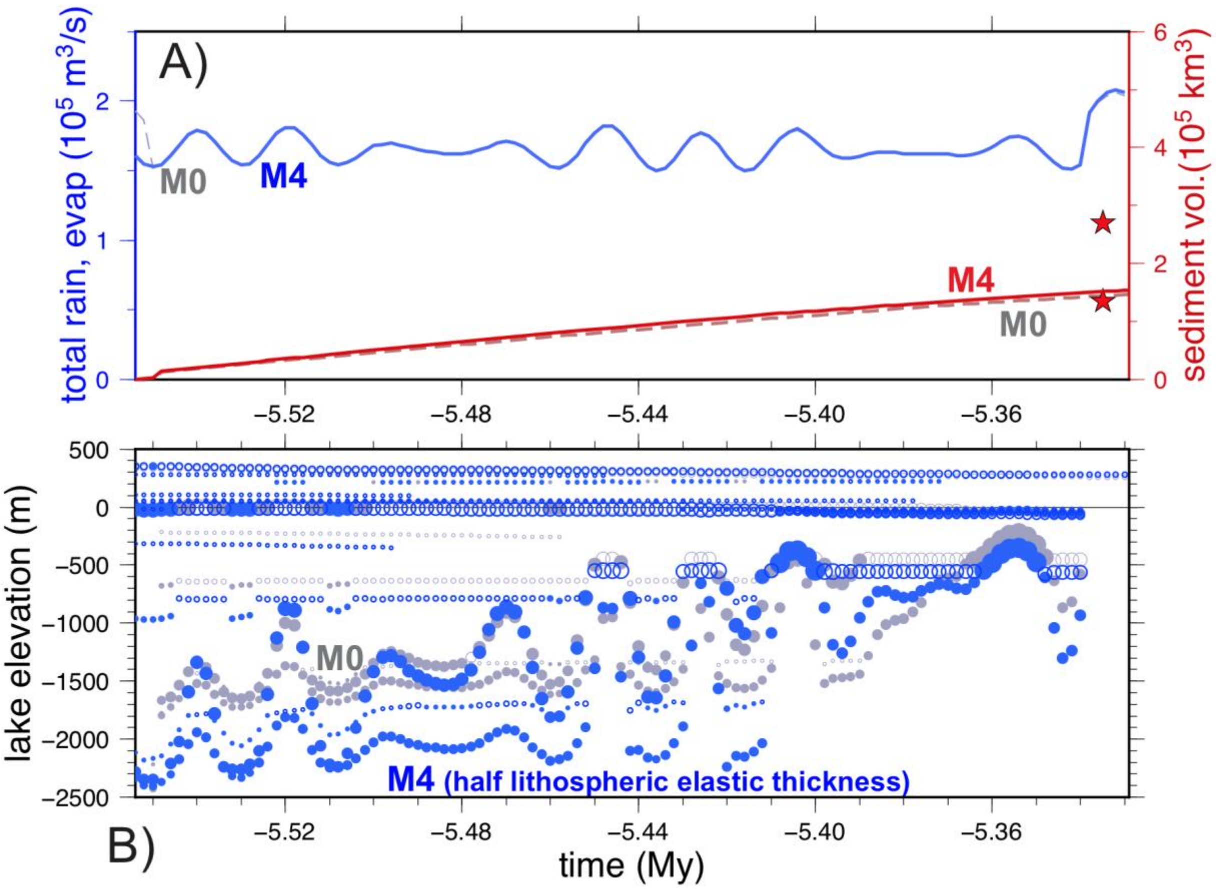

Fig. S1. Sensitivity of lake elevation evolution to the lithospheric elastic thickness. Results for a T_e of 10 km (M4, blue) compared to the reference model M0 (T_e=20 km). Legend as in Fig. 2. A) Evolution of the total precipitation (dark blue) and evaporation (light blue). B) Lake elevation evolution. Smaller T_e values predict deeper lake systems in the Mediterranean. We interpret that the drawdown during the MSC was enhanced by the low T_e values imposed by the Neogene tectonic rejuvenation of the Western Mediterranean lithosphere. The value adopted for the reference model (20 km) is a compromise between the lower values obtained in Neogene basins and the much older remnants of the Tethyan lithosphere in the E Mediterranean.

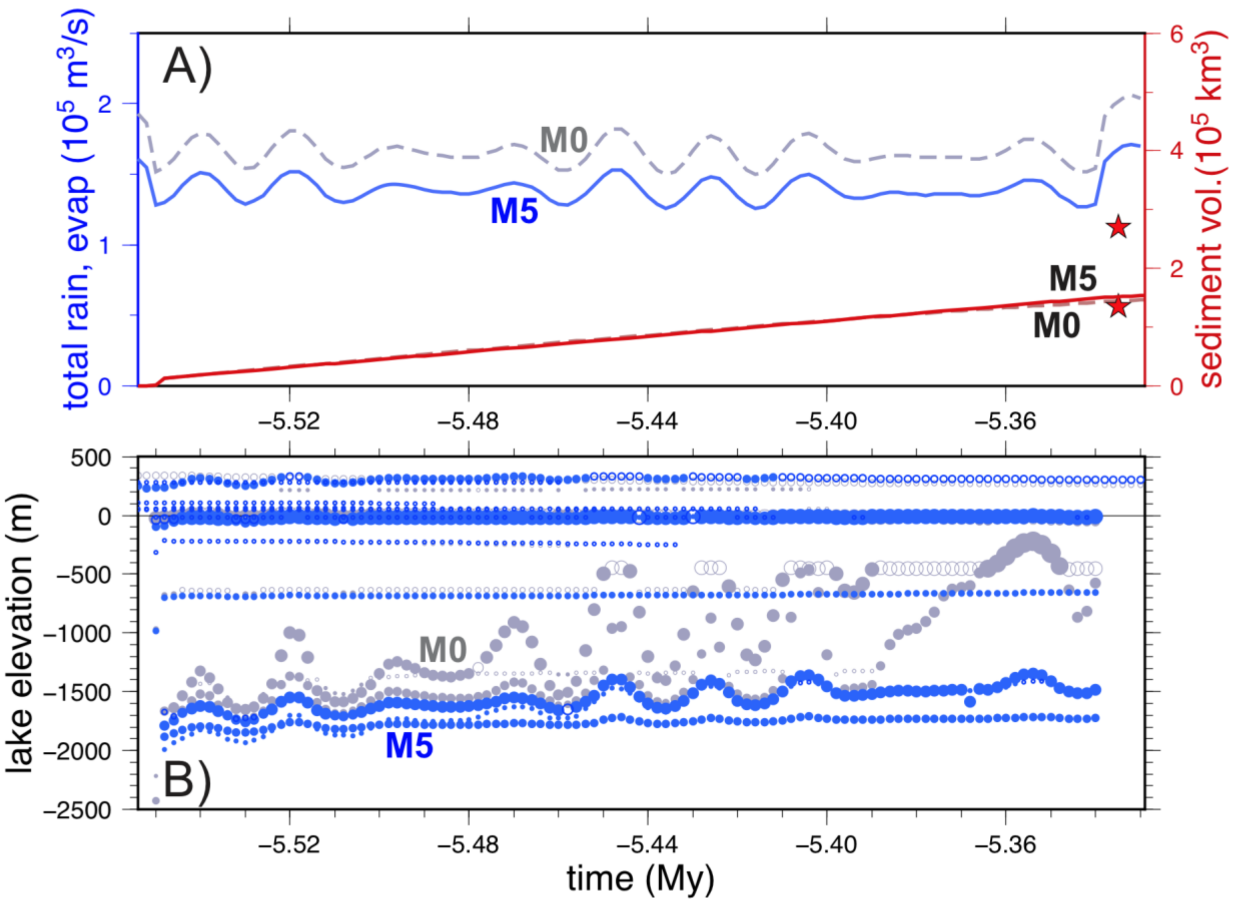

Fig. S2. Sensitivity of lake elevation evolution to precipitation. A) Precipitation and evaporation (blue) and the evolution of the sediment volume accumulated in the Mediterranean (red line), for model M5 (10% less precipitation *P*) and for the reference model M0 (grey dashed lines). B) Evolution of lake elevation for M0 (grey) and M5 (blue). Other legend as in Fig. 2. Lower precipitation leads to slightly more sedimentation because it increases the seafloor area exposed to subaerial erosion by making the lakes deeper and shallower. More importantly, it also leads to less outlet erosion, causing the water level of the main Mediterranean lakes to rarely rise above -1300 m.

Other Supplementary Materials for this manuscript include the following:

Movie S1.
Evolution of model M0 (reference model). Legend as in Figs. 1 and 2.

Movie S2.
Evolution of model M1 (less erosion). Legend as in Figs. 1 and 2.

Movie S3.
Evolution of model M2 (no precession cycles). Legend as in Figs. 1 and 2.
